# Evaluation of knowledge and awareness of invasive fungal infections amongst resident doctors in Nigeria

**DOI:** 10.11604/pamj.2020.36.297.23279

**Published:** 2020-08-18

**Authors:** Rita Oladele, Akaninyene Asuquo Otu, Olubunmi Olubamwo, Olufunmilola Bamidele Makanjuola, Ernest Afu Ochang, Joan Ejembi, Nicholas Irurhe, Iember Ajanaku, Halimat Ayodele Ekundayo, Adebola Olayinka, Oluwole Atoyebi, David Denning

**Affiliations:** 1 College of Medicine, University of Lagos, Lagos, Nigeria,; 2 Department of Internal Medicine, College of Medical Sciences, University of Calabar, Cross River State, Nigeria,; 3 Department of Public Health, University of Eastern Finland, Kuopio, Finland,; 4 College of Medicine, University of Ibadan, Oyo State, Nigeria,; 5 Department of Medical Microbiology, College of Medical Sciences, University of Calabar, Cross River State, Nigeria,; 6 Department of Medical Microbiology, Ahmadu Bello University Teaching Hospital, Zaria, Nigeria,; 7 University of Abuja Teaching Hospital, Abuja, Nigeria,; 8 Ilorin General Hospital, Ilorin, Kwara State, Nigeria,; 9 National Postgraduate Medical College of Nigeria, Abuja, Nigeria,; 10 The National Aspergillosis Centre, University Hospital of South Manchester, Manchester, United Kingdom

**Keywords:** Invasive fungal infections, Nigeria, resident doctors, continuing medical education

## Abstract

**Introduction:**

it has been estimated that about 11.8% of the Nigerians suffer serious fungal infections annually. A high index of suspicion with early diagnosis and institution of appropriate therapy significantly impacts on the morbidity and mortality of invasive fungal infections (IFIs).

**Methods:**

we conducted a cross-sectional multicentre survey across 7 tertiary hospitals in 5 geopolitical zones of Nigeria between June 2013 and March 2015. Knowledge, awareness and practice of Nigerian resident doctors about the diagnosis and management of invasive fungal infections were evaluated using a semi-structured, self-administered questionnaire. Assessment was categorized as poor, fair and good.

**Results:**

834(79.7%) of the 1046 participants had some knowledge of IFIs, 338(32.3%) from undergraduate medical training and 191(18.3%) during post-graduate (specialty) residency training. Number of years spent in clinical practice was positively related to knowledge of management of IFIs, which was statistically significant (p < 0.001). Only 2 (0.002%) out of the 1046 respondents had a good level of awareness of IFIs. Only 4(0.4%) of respondents had seen > 10 cases of IFIs; while 10(1%) had seen between 5-10 cases, 180(17.2%) less than 5 cases and the rest had never seen or managed any cases of IFIs. There were statistically significant differences in knowledge about IFIs among the various cadres of doctors (p < 0.001) as level of knowledge increased with rank/seniority.

**Conclusion:**

knowledge gaps exist that could militate against optimal management of IFIs in Nigeria. Targeted continuing medical education (CME) programmes and a revision of the postgraduate medical education curriculum is recommended.

## Introduction

Invasive fungal infections (IFIs) are life threatening infections caused by various types of fungal species [1]. The last two decades have recorded a significant increase in the global incidence of IFIs. This increase has been attributed to the human immunodeficiency virus (HIV) pandemic, medical and oncological therapeutic advances and the presence of better diagnostics for IFIs [2]. Other identified risk factors for IFI include malnutrition, severe burns, systemic corticosteroids for > 7 days, diabetes mellitus and multiple major surgery [3, 4]. However the high morbidity and mortality from IFIs has been linked to delayed diagnosis and treatment, the development of resistance and the severity of illness [5]. The commonest causative organisms of IFIs include *Candida spp, Cryptococcus neoformans, Aspergillus spp, Histoplasma capsulatum and Pneumocystis jirovecii* [1, 6, 7]. The estimated annual incidence of invasive mycoses due to some of these pathogens is > 400,000 infections per year with 30-95% mortality for Candida species, > 1,000,000 infections per year with 20-70% mortality for *C. neoformans*; > 200,000 infections per year with 30-95% mortality for Aspergillus species and > 400,000 infections per year with 20-80% mortality for *Pneumocystis jirovecii* [6]. In the African continent, the high prevalence of HIV/AIDS has provided substantial data on the burden of cryptococcal infections and *Pneumocystis jirovecii (carinii)* pneumonia (PCP) [8, 9]. There is however a paucity of data on invasive candidiasis, aspergillosis, mucormycoccosis, and other systemic mycotic infections like histoplasmosis.

Nigeria has an estimated population of 170 million people and 72,000 doctors registered with the Medical and Dental Council of Nigeria (MDCN). There is dearth of data on IFIs in Nigeria despite it being a high burden HIV and TB country [10]. New estimates from 2019 indicate a national HIV prevalence in Nigeria of 1.4% among adults aged 15-49 years [11]. A modelling study estimated that 11.8% of Nigerians suffer serious fungal infections annually [12]. Currently, there are only four licensed antifungal agents for IFIs (amphotericin B, fluconazole, itraconazole and voriconazole) in Nigeria and these are not readily accessible [13]. Liposomal amphotericin B, flucytosine, micafungin, caspofungin, anidulafungin, posaconazole and the recently approved (in Europe and the USA) isavuconazole are not available. Most of the routine laboratories in the country only utilize conventional diagnostic tests such as direct microscopy, histopathology and culture, but not immunodiagnostics or molecular detection techniques. This severely limits the capacity to effectively diagnose and treat IFIs, despite early diagnosis being a critical component for prognosis [5]. Given the dearth of clinical microbiologists and Infectious Diseases physicians in Nigeria today, it is doubtful that IFIs teaching is being prioritized in the medical training curriculum in Nigeria with attendant consequences for persons who develop IFIs. We set out to evaluate the knowledge and awareness of IFIs amongst resident doctors from all the geo-political zones in Nigeria via a multicentre survey.

## Methods

**Study design:** this was a cross-sectional multicentre survey evaluating Nigerian resident doctors´ knowledge, awareness and practice about pathogenic fungi, patients at risk, diagnosis and treatment of IFIs. We conducted this across 7 tertiary hospitals in 5 geopolitical zones of the country. The seven tertiary care hospitals that participated in the study were: Lagos University Teaching Hospital (LUTH) Lagos, Lagos State University Teaching Hospital (LASUTH), University of Calabar Teaching Hospital (UCTH) Calabar, University College Hospital, Ibadan (UCH), University of Ilorin Teaching Hospital (UITH) Ilorin, Ahmadu Bello University Teaching Hospital (ABUTH) Zaria and National Hospital Abuja. The study was conducted between June 2013 and March 2015. The study protocol was duly reviewed and national approval given by the ethics review committee of the Lagos University Teaching Hospital (LUTH). Resident doctors working in areas typically engaged with diagnosing and managing patients with IFIs were targeted (i.e. hematology, pathology, medical microbiology, oncology, internal medicine, paediatrics, surgical units, radiology and intensivists) and invited to participate in the survey. Participation in the survey was voluntary and verbal consent was sought in all cases. The consenting doctors were then issued a questionnaire which was self-administered on the spot. No incentives were provided to participating doctors and consultation of any medical literature (i.e. books, apps and websites) was discouraged. The specialties were grouped based on the structure of the faculties in the West African College of Medicine as follow: 1) internal medicine, public health, and family medicine; 2) paediatrics; 3) all surgical subspecialties including anesthesiology; 4) laboratory medicine; 5 others.

**Data collection and collation:** a semi-structured, self-administered questionnaire was used to retrieve data on knowledge and awareness of IFIs. It was divided into the following sections: general demographic (age, sex, rank, specialty and post- graduate years), knowledge/awareness and management practices regarding IFIs. It included several multi-stem true or false questions. Correct answers were determined by ROO, OOO, OO, OE and MOB and it was based on current international guidelines [14, 15]. Key operational definitions included: 1) IFIs: this term was used to describe severe, systemic infections with yeasts or molds. 2) Molds: this referred to multinucleated, filamentous fungi composed of branching tubular structures called hyphae. 3) Yeasts: this referred to eukaryotic, single-celled microorganisms with some species having the ability to develop multicellular budding cells known as pseudohyphae or false hyphae. 4) Dermatophytosis: this referred to fungal infections by dermatophytes (Trichophyton, Microsporum, and Epidermophyton) that infect keratinous tissue and are able to invade the superficial layers of hair, skin, and nails of a living host. Under the awareness section, the questions centred around organisms that caused IFI and risk factors for IFI. Under the management of IFI section, the questions bordered on diagnostic modalities for IFIs and the specific therapeutic options for various IFIs including medications used for prophylaxis. Each correct answer was scored as 1 point and each incorrect answer as 0 point. The questionnaire was pretested amongst ten resident doctors in medical microbiology at LUTH.

**Statistical analysis:** the questionnaires were reviewed and incomplete forms were omitted. The data was coded, entered and analysed using the Statistical Package for Social Sciences (SPSS) IBM version 21. The total number of items under assessment of awareness of IFIs was 26. Awareness of IFI was categorized as poor if < 11 (< 40%) was answered correctly, fair if between 11 and 18 (40-69%) were answered correctly and good if ≥ 19 (≥ 70%) were answered correctly. The total number of items under management of IFI was 38. Management of IFI was categorized as poor if ≤ 15 (< 40%) was answered correctly, fair if between 16 and 26 (40-69%) were answered correctly and good if ≥ 27 (≥ 70%) were answered correctly. The results were collated and summarized as percentages and proportions and represented in tables. Continuous variables were presented as means. Chi squared test was used to compare the differences across groups.

## Results

There were a total of 1046 respondents; 675(64.5%) were males while 367(35.1%) were females; with male to female ratio of 1.8: 1. Five hundred and Eighty-one (55.5%) of the respondents were within the age range of 31-40 years while those within the age range of 21-30 years constituted 38.4% of the respondents. The mean age of all the respondents was 32.36 ± 0.16. With respect to cadre, the junior resident doctors were in the majority (504; 48.2%) while the consultants were in the minority (6; 0.6%). Five hundred and eighty-two (55.6%) of the participants were from the surgical subspecialties (3), while only 70(6.7%) were specializing in laboratory medicine as shown in Table 1.

**Table 1:** characteristics of the respondents

Variable	Frequency	Percentage (%)
**Gender**		
Male	675	64.5
Female	367	35.1
Non response	4	0.4
**Total**	**1046**	**100.0**
**Age (years)**		
21-30	402	38.4
31-40	581	55.5
41-50	58	5.5
51 and above	5	0.5
**Total**	**1046**	**100.0**
**Rank**		
House Officer	236	22.6
Junior Resident	505	48.2
Senior Resident	259	24.8
Medical Officer	30	2.9
Consultant	6	0.6
Non response	11	1.1
**Total**	**1046**	**100.0**
**Specialties**		
1	218	20.9
2	78	7.5
3	582	55.6
4	70	6.7
5	98	9.4
**Total**	**1046**	**100.0**

The specialties of the respondents were grouped using as follow: 1. Internal medicine, public health, and family medicine; 2. Pediatrics; 3. All surgical subspecialties including anesthesiology; 4. Laboratory medicine; 5. others

In relation to knowledge of IFIs; 834(79.7%) of the respondents had some knowledge of IFIs. The commonest source of knowledge about IFIs was from undergraduate training in medical school 338 (32.3%) followed by 303(29.0%) from personal reading while others 191(18.3%) learnt about IFIs during the course of their post-graduate residency training. The ability to identify common IFIs appeared to be high as 813(77.7%) and 820(78.4%) were able to correctly identify invasive aspergillosis and invasive candidiasis as IFIs respectively. The level of non-response to these two questions was similar (18.4% and 18.1%). When asked to identify organisms that cause IFIs; *Fusarium spp, Candida spp, Aspergillosis spp, Mucor spp* and *Cryptococcus spp* were correctly identified by 52.4%, 69.7%, 67.9%, 20.6%, and 62.3% respectively. There were varied responses with regards to the best method of diagnosing IFIs. While 324 (31%) agreed that the diagnosis of IFIs could be based on a combination of clinical and laboratory parameters; 40 (3.8%) of respondents did not think that the diagnosis should be based on several criteria (Table 2). Only 4(0.4%) of respondents had seen more than ten cases of IFIs; while 10(1%) had seen 5-10 cases, 180(17.2%) < 5cases and the rest had never seen or managed any cases of IFIs. Respondents were more likely to diagnose yeast than mold infections (50.4% vs 16.1%). Five hundred and eighty-eight (56.2%) of the respondents would not base their treatment of IFIs on laboratory tests while 616 (58.9%) of them would embark on empirical treatment with antifungals in suspected cases of cases IFIs. Fluconazole was identified as the ideal antifungal of choice for pre-emptive therapy against *Aspergillus* by 442(42.3%) of the respondents; and for Candida, fluconazole was again the commonest choice among 599(57.3%) of the respondents (Table 3).

**Table 2:** methods of diagnosing invasive fungal infections

How best do you believe IFIs should be diagnosed	Yes frequency (%)	No frequency (%)	No response frequency (%)
**Clinically**	534(51.1)	75(7.2)	437(41.8)
**Histology**	511(48.9)	86(8.2)	449(42.9)
**Culture**	473(45.2)	56(5.4)	517(49.4)
**Serology**	347(33.2)	80(7.6)	619(59.1)
**Polymerase chain reaction**	206(19.7)	45(4.3)	795(76.0)
**Combination of above**	324(31.0)	40(3.8)	672(65.2)

**Table 3:** prophylaxis of fungal infections

Choice of pre-emptive therapy	Yes Frequency (%)	No Frequency (%)	No response Frequency (%)
Aspergillus			
Fluconazole	442 (42.3)	56(5.4)	548(53.4)
Amphotericin B deoxycholate	183(17.5)	113(10.8)	750(71.7)
Liposomal amphotericin	114(10.9)	118(11.3)	814(77.8)
Echinocarndins	164(15.7)	126(12.0)	756(72.3)
Voriconazole	73(7.0)	124(11.9)	849(81.2)
**Candida**			
Fluconazole	599(57.3)	41(3.9)	406(38.8)
Amphotericin B deoxycholate	124(11.8)	169(16.2)	753(72.0)
Liposomal amphotericin	160(15.3)	105(10.0)	781(74.6)
Echinocarndins	154(14.8)	111(10.6)	781(74.6)
Voriconazole	130(12.4)	91(8.7)	825(78.8)

There were statistically significant differences in knowledge about IFIs among the various cadres of doctors (p < 0.001) as level of knowledge increased with rank/seniority. There were also statistically significant differences in participants´ perceived knowledge among the various specialties with the highest proportions coming from doctors in Anesthesiology and Ophthalmology 17(94.4%). The lowest proportions were recorded among doctors in Public Health 16(61.5%) and Radiology 23(67.6%) (Table 4). A statistically significant difference was also observed with respect to suspicion of IFI among the various specialties. Higher proportions of doctors who suspected IFI during their medical practice were recorded among doctors from internal medicine 87(64.0%) and anesthesiology 10(62.5%). The lowest proportions were recorded among doctors in general surgery 45(44.6%) and radiology 16(48.5%) (Table 5). There was a weak positive correlation between the age (p < 0.001) and rank (p = 0.004) of the respondents with the awareness and management of IFIs. The years spent in clinical practice showed a negative correlation with awareness of IFI but this relationship was not statistically significant (p = 0.858). However, the years spent in clinical practice showed a weak positive relationship with knowledge of management of IFIs and was statistically significant (p < 0.001). Awareness of IFIs among the respondents was strongly positively correlated with management of IFIs and this was statistically significant (p < 0.001) (Table 6). The rate of non-response for the all knowledge based questions was high and this was most probably due to poor or no knowledge of the subject matter. Overall, the awareness of IFI was low across the centres that participated in this survey as only 2 (0.002%) out of the 1,046 respondents scored ≥ 27 (≥ 70%).

**Table 4:** knowledge of invasive fungal infections by rank and specialty

	Do you know about Invasive fungal infection?		X2	df	P-value
	Yes	No			
**Rank of respondents**			47.094	4	0.000**
Senior registrar	205(88.4)	27(11.6)			
Junior registrar	423(86.9)	64(13.1)			
House officer	169(69.0)	76(31.0)			
Medical officer	25(92.6)	2(7.4)			
Consultant	6(100.0)	0(0.0)			
**Total**	**828(83.0)**	**169(17.0)**			
**Specialty of respondents**			68.080	12	0.000**
General Surgery	90(82.6)	19(17.4)			
Public health	16(61.5)	10(38.5)			
Internal medicine	131(89.1)	16(10.9)			
Family medicine	89(89.1)	10(10.1)			
Paediatrics	126(85.7)	21(14.3)			
O&G	116(82.3)	25(17.7)			
Pathology/ Lab medicine	106(92.2)	9(7.8)			
Anaesthesiology	17(94.4)	1(5.6)			
ENT	51(78.5)	14(21.5)			
Dentistry	0(0.0)	4(100.0)			
Ophthalmology	17(94.4)	1(5.6)			
Radiology	23(67.6)	11(32.4)			
Others	8(50.0)	8(50.0)			
**Total**	790(84.1)	149(15.9)			

**Table 5:** suspicion of invasive fungal infections by rank and specialty

	In your experience on patient management, do you ever suspect invasive fungal infection?		X2	df	P-value
	Yes	No			
**Rank of respondents**			**51.369**	**4**	0.000**
Senior registrar	149(68.7)	68(31.3)			
Junior registrar	245(53.7)	211(46.3)			
House officer	81(36.7)	140(63.3)			
Medical officer Consultant	18(62.1) 6(100.0)	11(37.9) 0(0.0)			
**Total**	**499(53.7)**	**430(46.3)**			
**Specialty of respondents**			**24.456**	**12**	0.018**
General Surgery	45(44.6)	56(55.4)			
Public health	13(50.0)	13(50.0)			
Internal medicine	87(64.0)	49(36.0)			
Family medicine	60(61.9)	37(38.1)			
Paediatrics	74(53.6)	64(46.4)			
O&G	65(48.5)	69(51.5)			
Pathology/ Lab medicine	68(60.2)	45(39.8)			
Anaesthesiology	10(62.5)	6(39.8)			
ENT	27(46.6)	31(53.4)			
Dentistry	0(0.0)	4(100.0)			
Ophthalmology	8(53.3)	7(46.7)			
Radiology	16(48.5)	17(51.3)			
Others	5(33.3)	10(66.7)			
**Total**	**478(54.0)**	**408(46.0)**			

**Table 6:** correlation of age, years of practice, and rank with awareness and management of invasive fungal infections

Awareness of invasive fungal infections	Spearman´s rank correlation	P-value
**Age**	0.117	0.000
**Years of practice**	-0.006	0.858
**Rank**	0.090	0.004
**Management of invasive fungal infections**	**Spearman´s rank correlation**	**P-value**
**Age**	0.172	0.000
**Years of practice**	0.145	0.000
**Rank**	0.049	0.116
**Awareness of IFI**	0.472	0.000

## Discussion

Our survey confirms the existence of knowledge gaps in awareness, knowledge and management of IFIs among resident doctors across Nigeria. This finding may be linked to the paucity of data on IFIs from sub-Saharan Africa and in Nigeria in particular. A study commissioned by International Society for Human and Animal Mycology (ISHAM) in 2000 to identify training gaps in developing countries reported that “the extent of diagnostic services in mycology in West Africa, based on questionnaire responses, showed that microscopy and culture were available in Nigeria and Gambia but serological tests were not and antifungal testing was routinely performed only in Gambia [16]. The ISHAM study also evaluated the publication trends on fungal diseases in Nigeria over the last 10 years and found that 44 of the 65 publications were on oral and vulvovaginal candidiasis, dermatophytes and African histoplasmosis and did not reflect IFIs. Strikingly, a 10-year retrospective study of mycological infections at a major tertiary hospital in Nigeria revealed no laboratory diagnosis of an IFI was made throughout the study period [17]. Unfortunately, this lopsided nature of things appears to have persisted in Nigeria.

Amongst those that claimed knowledge of IFIs; rank was statistically significant (p < 0.001) with majority being senior registrars (88.4%) and consultants (100%). However, when asked about whether they ever entertained the suspicion of IFIs in their routine practice it was mainly consultants (100%) and some senior registrars (68.7%) that had. Although majority (582; 55.6%) of the respondents were from surgical subspecialties, only doctors from anesthesiology appeared to have a fair awareness of IFIs (Table 4, Table 5). This is not surprising because in the Nigerian setting, intensive care units (ICU) are managed by anaesthesiologists. However, surgery remains a known risk factor for IFIs. Over fifty percent of the respondents stated that they would rely on laboratory reports for diagnosis of IFIs, favoring histology, culture or serology. This suggests that most of the respondents consider the clinical features of IFIs to be unreliable. Interestingly, amongst the doctors in Laboratory medicine, only 60% would routinely suspect IFIs, suggesting a major knowledge gap challenge which is likely to impact upon the outcome of persons with IFIs.

Whereas international practice guidelines (at the time of study) advocate for the use of fluconazole and voriconazole to treat *Candida* and *Aspergillus* infections respectively [14, 15], 57.2% and 42.3% of respondents opted for fluconazole as drug of choice for *Candida* and *Aspergillus* infections respectively. Such practice is likely to result in poor treatment outcomes as fluconazole is not active against invasive *Aspergillosis*. The knowledge gap demonstrated in this survey is concerning given that Nigeria has a Postgraduate Medical College of Medicine that trains resident doctors and awards fellowships. It is imperative that awareness of IFIs be developed so that IFIs are considered in the list of differential diagnosis especially among at-risk groups. In reality, the index of suspicion of IFI is usually low and the diagnosis is delayed or completely missed [18].

A cross-sectional cohort study in Europe showed that systemic antifungal therapy was administered to 7% of all patients admitted to an ICU, with only one-third of them having a documented IFI [19] and previous European studies demonstrating that inappropriate use of antifungal drugs may reach 67-74% in tertiary care hospitals [20]. Physicians in Europe have been reported to have problems differentiating colonization from infection when *Candida spp*. is isolated in urine or in a tracheal aspirate, which could lead to an over-prescription of antifungals [20]. Although it is important to entertain the diagnosis of IFIs especially in situations where the risk factors for IFIs are present, it is crucial to collect specimens for laboratory diagnosis of IFIs prior to commencement of antifungal therapy. It is also vital to make a distinction between colonization, superficial mycoses and IFIs.

In recent times, postgraduate training programmes have made concerted efforts to provide accurate, assessments of the competence of resident doctors. Such assessments are targeted at optimizing the capabilities of residents by providing motivation and direction for future learning [21]. In preventing IFI-related management errors, it is important to appreciate that the awareness of the burden of a disease condition and knowledge of its etiology, diagnosis and appropriate treatment, are related in a causal loop [22]. General awareness of the burden of IFI among physicians should influence the proportion of patients having IFI that will have a timely diagnosis or the probability that a physician will suspect IFI in relevant situations. Physician awareness together with Physician experience is likely to increase the probability of diagnosing IFIs where relevant. This will improve overall diagnostic accuracy, which is crucial for successful treatment. One limitation of this study is that we included mainly resident doctors in tertiary hospitals who are more likely to influence initial diagnoses and treatment. However, their opinions may not mirror that of the consultant physicians who have the duty to ratify interim treatment plans. Another limitation stemmed from the fact that some of the residents who participated were in supernumerary posts so we could not attribute the level of knowledge displayed to the centre of training.

## Conclusion

The knowledge gaps among medical doctors across Nigeria with respect to IFIs need to be urgently addressed. These gaps can be filled through improved and targeted continuing medical education (CME) programs in Nigeria and a review of the postgraduate medical curriculum.

### What is known about this topic

- Invasive fungal infections (IFIs) are life threatening infections caused by various types of fungal species;
- There is dearth of data on IFIs in Nigeria despite it being a high burden HIV and TB country;
- General awareness of the burden of a disease among physicians should influence the proportion of patients with that disease that will have a timely diagnosis and receive appropriate treatment.

### What this study adds

- There was a low level of knowledge of IFIs among the majority of the 1046 doctors sampled across Nigeria. Doctors with greater years of practice experience had better knowledge of management of IFIs;
- The knowledge gaps demonstrated among doctors across Nigeria with respect to IFI is profound and is likely to impact negatively on patients with these serious clinical conditions;
- Targeted continuing medical education (CME) programmes and a revision of the postgraduate medical education curriculum is recommended.
